# 3D laser nano-printing on fibre paves the way for super-focusing of multimode laser radiation

**DOI:** 10.1038/s41598-018-32970-6

**Published:** 2018-10-02

**Authors:** Grigorii S. Sokolovskii, Vasileia Melissinaki, Ksenia A. Fedorova, Vladislav V. Dudelev, Sergey N. Losev, Vladislav E. Bougrov, Wilson Sibbett, Maria Farsari, Edik U. Rafailov

**Affiliations:** 1 Ioffe Institute, 26 Polytechnicheskaya str., St. Petersburg, 194021 Russia; 2 ITMO University, 49 Kronverksky pr., St. Petersburg, 197101 Russia; 3 IESL-FORTH, N. Plastira 100, 70013 Heraklion, Greece; 4 Department of Physics, University of Crete, 71003 Heraklion, Greece; 5 Aston Institute of Photonic Technologies, Aston University, Aston Triangle, Birmingham, B4 7ET UK; 6 St. Petersburg State Electrotechnical University (LETI), 5 Prof. Popova str., St. Petersburg, 197022 Russia; 7 School of Physics and Astronomy, University of St. Andrews, St. Andrews, Fife, Scotland KY16 9SS UK

## Abstract

Multimode high-power laser diodes suffer from inefficient beam focusing, leading to a focal spot 10–100 times greater than the diffraction limit. This inevitably restricts their wider use in ‘direct-diode’ applications in materials processing and biomedical photonics. We report here a ‘super-focusing’ characteristic for laser diodes, where the exploitation of self-interference of modes enables a significant reduction of the focal spot size. This is achieved by employing a conical microlens fabricated on the tip of a multimode optical fibre using 3D laser nano-printing (also known as multi-photon lithography). When refracted by the conical surface, the modes of the fibre-coupled laser beam self-interfere and form an elongated narrow focus, usually referred to as a ‘needle’ beam. The multiphoton lithography technique allows the realisation of almost any optical element on a fibre tip, thus providing the most suitable interface for free-space applications of multimode fibre-delivered laser beams. In addition, we demonstrate the optical trapping of microscopic objects with a super-focused multimode laser diode beam thus rising new opportunities within the applications sector where lab-on-chip configurations can be exploited. Most importantly, the demonstrated super-focusing approach opens up new avenues for the ‘direct-diode’ applications in material processing and 3D printing, where both high power and tight focusing is required.

## Introduction

Since their demonstration and development in 1970–80s^1,2^, optical tweezers have become one of the key laser-based assets in physics and biology for the contact-free manipulation of microscopic objects, through the harnessing of forces that originate from tightly-focused laser beams. By focusing a laser beam to a diffraction-limited spot near a micrometer-sized living cell or a dielectric particle, a strong light gradient in the vicinity of the focus induces the necessary trapping/manipulation force. The balanced combination of the gradient force with a light-scattering force and gravity thus enables the conditions that satisfy stable optical trapping. Dynamic stability is facilitated by the viscosity of the fluid medium, which damps the oscillations.

Manipulation of cells using the contact-free and damage-free advantages of optical tweezing makes this approach compatible with the requirements involved in optical sorting of mixed colloidal particles and living cell populations having ranges of sizes and refractive indices^3,4^. It is impressive that optical tweezing techniques have been used successfully to probe the cytoskeleton^5^, to study cell motility^6^, and have enabled the targeted delivery of nanoparticles into a specified region of the interior of individual living cells^7^. Optical tweezers have also been shown to be well suited to assessments aimed at resolving the step-like motions of motor proteins such as kinesin^8^ and myosin^9^, and for demonstrating that such motors can be controlled to advance for hundreds of steps^10^. It has to be appreciated that these techniques and their introduction into day-to-day applications have been hindered by the involvement of expensive laser systems, whereas in contrast, semiconductor lasers offer unparalleled compactness and integrability together with high efficiency and direct electrical control. Employing laser diodes for optical trapping and tweezing could thus be expected to have a profound impact on the further progress of this technology.

By developing a solution to the beam-quality issue of diode lasers by employing appropriate beam shaping techniques, such as Bessel beam generation using an axicon, it can be envisaged that diode-laser-based optical manipulation could be rendered as a day-to-day and relatively cost-effective modality within medical biophotonics.

## Super-focusing of beams with high propagation parameters M^2^

Wide-aperture semiconductor lasers, although ideal tools for a number of higher-power applications, suffer from filamentation and multi-mode generation. This makes it nearly impossible to use this type of lasers when a tightly focused laser beam is necessary, e.g. for direct material processing, 3D laser printing or in beam traps. Typically, the beam quality of a laser beam is described by the beam propagation factor *M*^2^ ^11,12^ (also called “beam quality factor”). The *M*^2^ value is typically derived as a fraction with the divergence of the given beam as a numerator and the Gaussian beam divergence as a denominator. This definition means that the ‘ideal’ Gaussian beam with minimal divergence (limited by diffraction) will have *M*^2^ = 1. Similarly, *M*^2^ gives the factor of magnification of the beam waist of a multi-mode laser beam comparing to the ‘ideal’ Gaussian counterpart produced with the same focusing optics. This allows very convenient introduction of a ‘quasi’ Gaussian beam, which is ‘almost’ Gaussian in the far field but wider in the focal plane. Mathematically, this convenience is allowed by a numerical *M*^2^-fold increase of the wavelength in the mathematical description of the Gaussian beams. Typically, high-power laser diodes have *M*^2^-factor values in the range of 20 to 30. Therefore, the quasi-Gaussian beams emitted by multimode semiconductor lasers have spot sizes that exceed Gaussian-beam spot sizes by one to two orders of magnitude.

**Figure 1:**
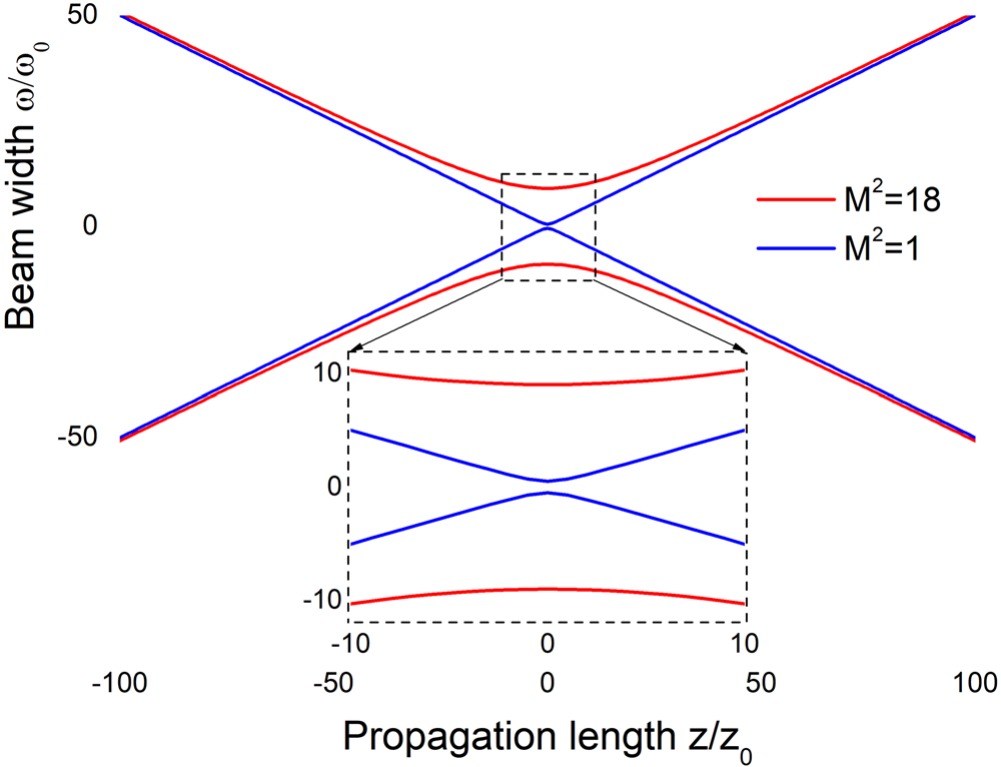
Calculated beam width ω along *z* axis for a Gaussian beam (red) with a unity beam propagation factor and for a quasi-Gaussian counterpart (blue) with the beam propagation factor of 18. The graph is scaled to the ideal Gaussian Rayleigh range *z*_0_ and its focal spot size ω_0_.

In Fig. 1, the calculated path of a beam width ω is shown along the *z* axis for two beams: a Gaussian beam with a unity beam propagation factor and a quasi-Gaussian beam with the beam propagation factor of 18. The graph is scaled to the ideal Gaussian Rayleigh range *z*_0_ and its focal spot size ω_0_. This figure demonstrates that the focusing of multiple mode laser diode beams can be a substantial problem that severely limits the use of high-power semiconductor lasers in applications where tight focusing is required.

At the moment, low-cost laser sources are spreading into many fields of science and technology and many techniques have been suggested to improve their multi-mode output pattern. Most of these, however, rely on manipulation with the laser phase front and/or compensation of the wavefront ‘scrambling’ by the multimode optical fibre. This is typically achieved by the spatial light modulators (SLMs) or deformable mirrors (DMs)^13–15^, which add significant costs, complexity and footprint to the setups involving low-cost laser sources and multimode fibres. The approach presented in this paper therefore stands out as utilizing a single ultra-compact (and potentially very low-cost and mass-producible) element integrated to the optical fibre.

The concept of ‘super-focusing’ for laser-diode high-*M*^2^ beams has been recently proposed and demonstrated experimentally by generating a Bessel beam using a conical lens^16^. This type of non-diffracting beams (i.e. able to maintain its intensity during propagation)^17–19^ is so-called because its amplitude profile has the form of a zero-order Bessel beam. Such a beam can be created by a plane wave (or collimated Gaussian beam) passing through a conical lens (so-called axicon). The diameter of the central spot of the Bessel beam is determined by the apex angle of the axicon and can be on the scale of an optical wavelength. As expected, traditional focusing methods with multimode radiation lead to a range of wavefront curvatures relating to the constituent modes and this in turn gives rise to shifts in their focal points along the optical axis. This results in large focal-spot sizes with increased *M*^2^ values. By contrast, Bessel beam generation using an axicon depends on ‘self-interference’ of each mode, thereby eliminating any increase in the focal-spot size.

The transverse size of the central spot of the Bessel beam, which is created after the propagation of a collimated multimode quasi-Gaussian beam through an axicon, considerably increases with propagation distance due to a divergence of the initial beam^20^. This, together with the axicon aperture, places physical limits on the propagation length of the resulting Bessel beam. Fortunately, the initial diameter of the central lobe of the Bessel beam can be few-fold smaller comparing to the minimal focal spot size of a Gaussian beam with high a *M*^2^ value.

It is possible to define a figure of merit (FOM) for such ‘super-focusing’ of a multi-mode quasi-Gaussian beam based on self-interference of the constituting modes. This expresses as a regular fraction with the numerator given by the minimal focal spot size of a high *M*^2^ quasi-Gaussian beam ω_min_ = *M*^2^λ/π and the denominator being the minimal central lobe radius *r*_0_ of the Bessel beam produced with an axicon of refractive index *n* and apex angle *α* from the given quasi-Gaussian beam:

1 $$FOM=\frac{{\omega }_{{\rm{\min }}}}{{r}_{0}}\approx {M}^{2}(n-1)\cos \,\tfrac{\alpha }{2}$$

**Figure 2:**
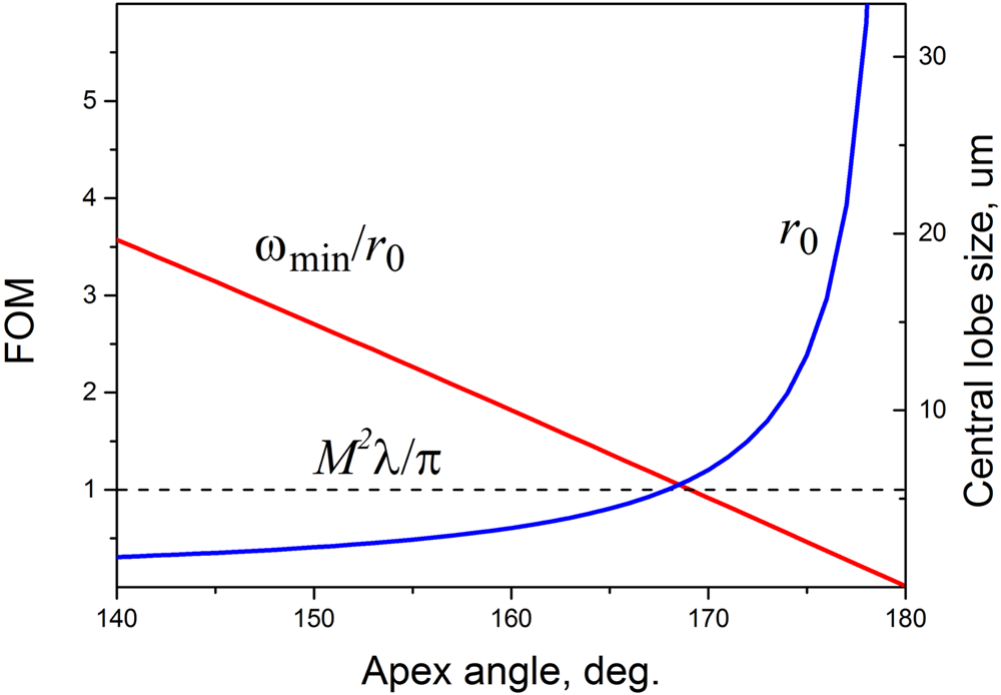
Figure of merit (FOM) for the super-focusing of a high-*M*^2^ quasi-Gaussian beam as a function of the apex angle of the axicon (red). The graph also shows the minimal central lobe radius *r*_0_ of the Bessel beam (blue) and the minimal focal spot size (dotted line) of a high *M*^2^ quasi-Gaussian beam of wavelength λ = 960 nm and beam propagation factor *M*^2^ = 18.

In Fig. 2 the FOM is shown for the super-focusing of a high-*M*^2^ quasi-Gaussian beam as a function of the apex angle of the axicon. The graph also shows the minimal central lobe radius *r*_0_ of the Bessel beam and the minimal focal spot size of a high *M*^2^ quasi-Gaussian beam of 960 nm wavelength and beam propagation factor of 18 (the last does not depend on the axicon angle and therefore is constant). It is clearly evident that, by employing the ‘self-interference’ focusing, about two-fold super-focusing can be achieved with a 160° axicon and more than a 3.5-fold super-focusing with a 140° axicon.

## Experimental Assessments

In results already published, it has been demonstrated that optical trapping is feasible with axicon-based Bessel beams generated from semiconductor lasers^21^. For this present assessment, we fabricated an axicon on an optical fibre, and we used it to super-focus a high-*M*^2^ laser diode beam, which we subsequently employed in optical trapping. This approach to beam-shaping, based on axicon geometry that is fabricated on an optical fibre, can thus offer unparalleled compactness and integration possibilities.

When considering super-focusing a high-*M*^2^ beam with an axicon, it is crucial to take into account the radius of its apex. Typically, this radius is around 100 µm, though the diameter of rounding at the tip of the high-end axicon may even be <50 µm so its effect is negligible for most practical applications. Also, the effect of rounding of the axicon tip can be almost completely avoided by the appropriate blocking of the affected part of Bessel beam^22^. However, when one considers superfocusing, even small imperfection of the axicon may significantly increase the size of the central lobe of the output beam^20^. Therefore, the quality of the axicon apex is crucial for practical realisation of the super-focusing technique.

The micro-axicons investigated in this research were fabricated by 3D laser nano-printing, also termed as multi-photon lithography (MPL)^23,24^, on the edge of a 100 µm core optical fibre. This is a laser-based additive manufacturing technique, which allows the direct fabrication of fully 3D microstructures with resolutions beyond the diffraction limit. When the beam of a sub-picosecond laser is tightly focused inside the volume of a transparent, photo-structurable polymer, the high intensities within the beam voxel can result in multi-photon absorption, thereby causing the local photo-polymerisation of the material. By translating the laser beam within the material, 3D structures can be directly “written” and then, by afterwards removing the unpolymerised photopolymer using an appropriate chemical developer, one is left with the desired 3D structure.

**Figure 3:**
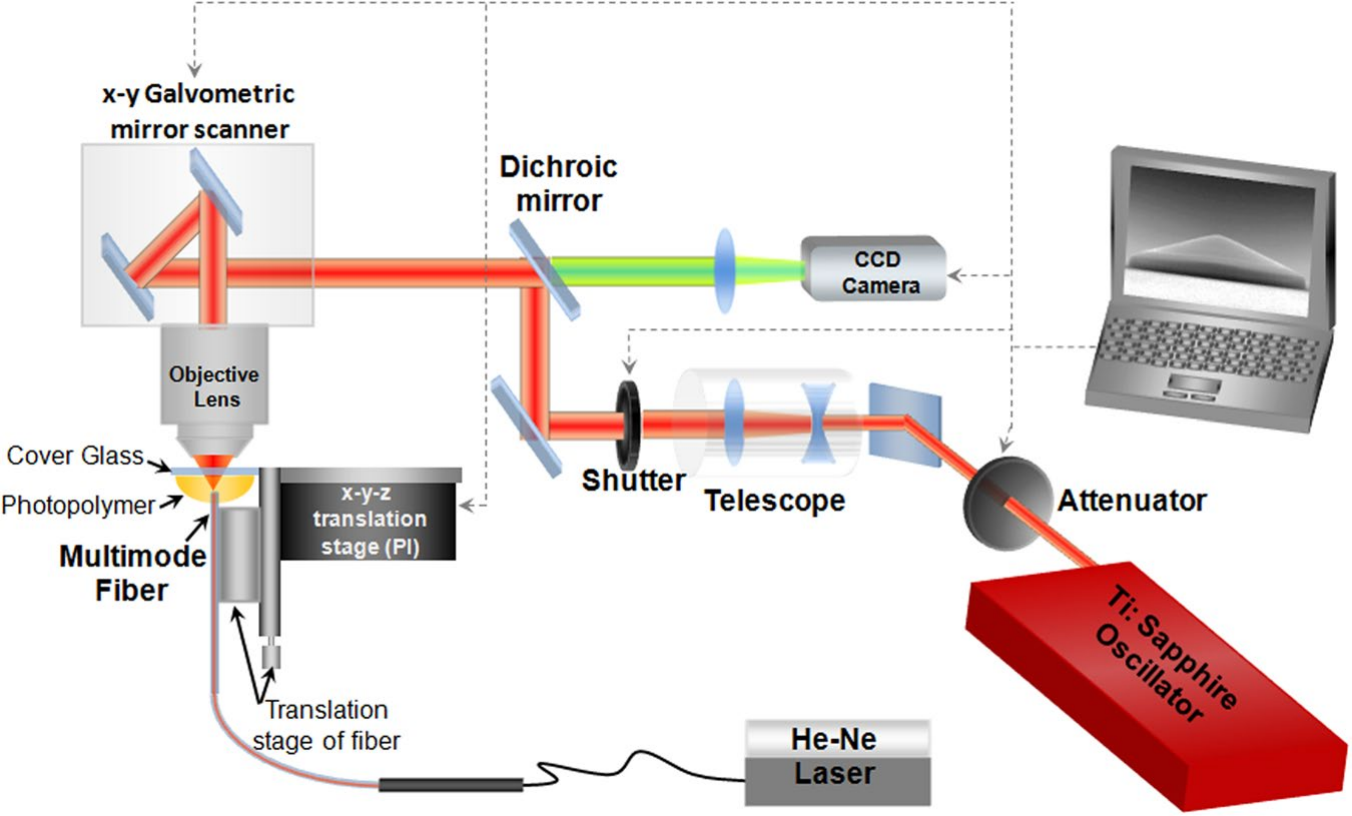
Experimental setup that is specially adapted for the direct laser writing on fibre.

**Figure 4:**
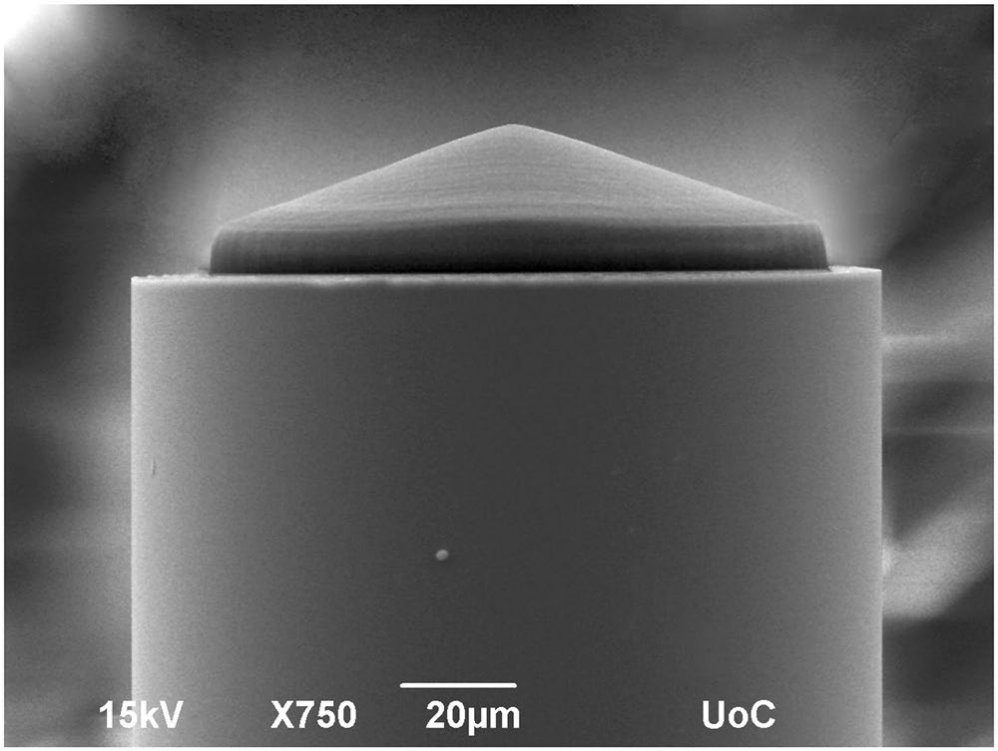
Scanning electron microscope photograph of a micro-axicon on a tip of a 100 μm optical fibre. The axicon apex angle is 140°. The transverse size of the rounded apex area is below 10 μm.

The experimental 3D laser nano-printing set-up that was specially adapted for the fabrication on a fibre-end^25,26^, is shown schematically in Fig. 3. Details of the 3D laser nano-printing of axicon on fibre are given in the Methods. The resulting size of the rounded apex area was below 10 µm for all 140° and 160° micro-axicons used in our experiments (see Fig. 4). The contrary side of the optical fibre was polished and terminated with the standard fibre coupler. This was done to suppress distortions of the wavefront, which are typical for as-cleaved optical fibres.

Similar technique of 3D laser nano-printing on the single-mode fibres^27^ and SMF cables^28^ enables fabrication of complex micro-optics including high-performing multi-lens elements, free-form surfaces and photonic crystals. However, the complex approach of ^27,28^ is limited to the single-mode radiation and is not acceptable for high-power (multi-mode) applications, thus offering no solution for focusing of high-M^2^ beams. One can also envisage utilization of less sophisticated and relatively easy-to-handle single-photon 3D microfabrication techniques^29,30^ for fabrication of the fibre-based micro-optics. In future, these may enable mass production of low-cost tiny optical elements. However, at the moment single-photon 3D laser nano-printing is obviously less mature comparing to the multi-photon counterpart.

**Figure 5:**
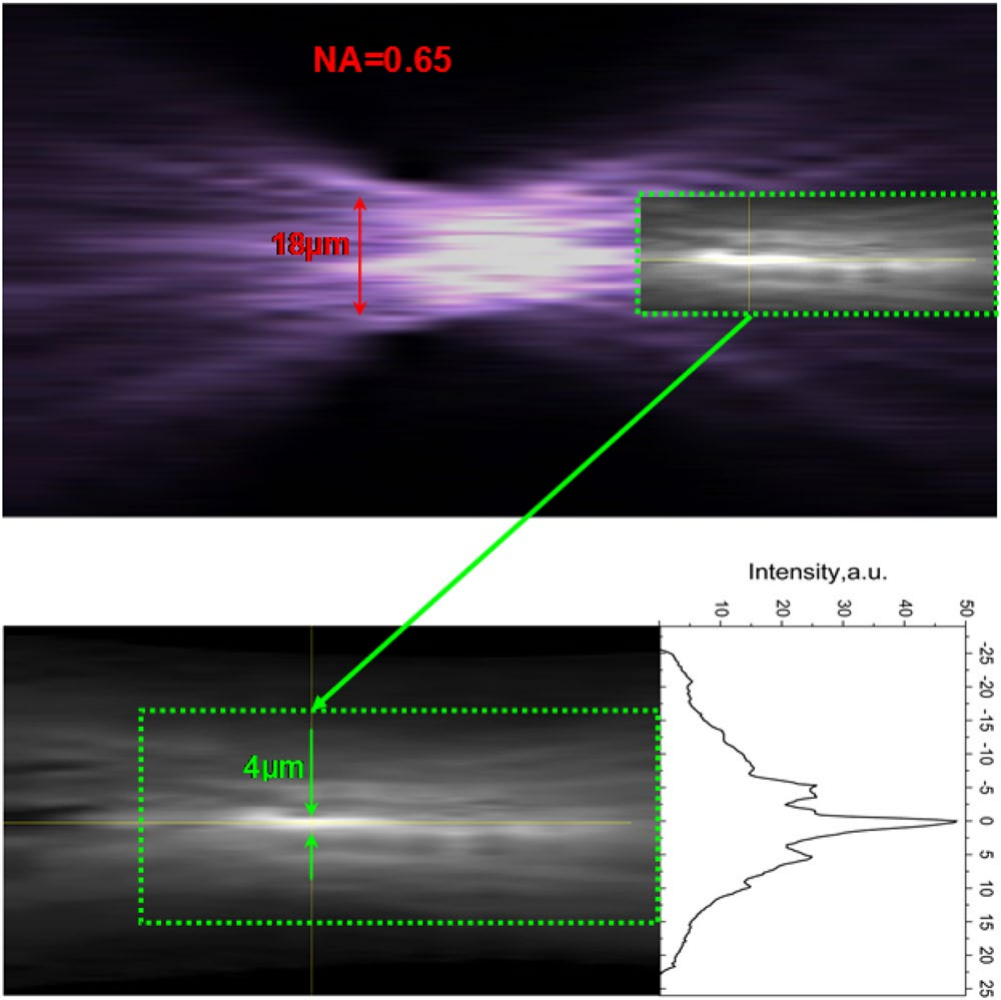
Focusing of a beam of a semiconductor laser with NA = 0.65 lens (top figure) and superfocusing of the same beam with a 140° axicon fabricated on the tip of a 100 μm optical fibre (bottom figure). The transverse size of the super-focused ‘needle’ beam with an approximately 20 μm propagation length was 2–4 μm. This gives approximately an order of magnitude reduction of the focal spot size in comparison to the minimal focal spot size of ~11 μm achievable with an ideal NA = 1 lens for a laser beam of 960 nm wavelength and beam propagation parameter of 18.

In our experiments we used the laser diode with a beam propagation factor of 18 and 960 nm output wavelength. A typical axial image of the laser output super-focused with an 140° micro-axicon is shown in Fig. 5. This figure also shows the axial distribution of the same laser pattern focused by NA = 0.65 objective lens. For the given laser beam parameters, the minimal focal spot size given by relation 2ω_min_ = 2*M*^2^λ/π is approximately 11 μm. This agrees well with the measured focal spot of 18 µm for NA = 0.65 lens in Fig. 5. In this figure, the transverse size of the needle-beam produced with the 140° micro-axicon is demonstrated to be approximately 2–4 µm with a propagation length of 20 µm. This gives approximately an order of magnitude reduction of the focal spot size in comparison to the minimal focal spot size achievable with an ideal NA = 1 lens.

**Figure 6:**
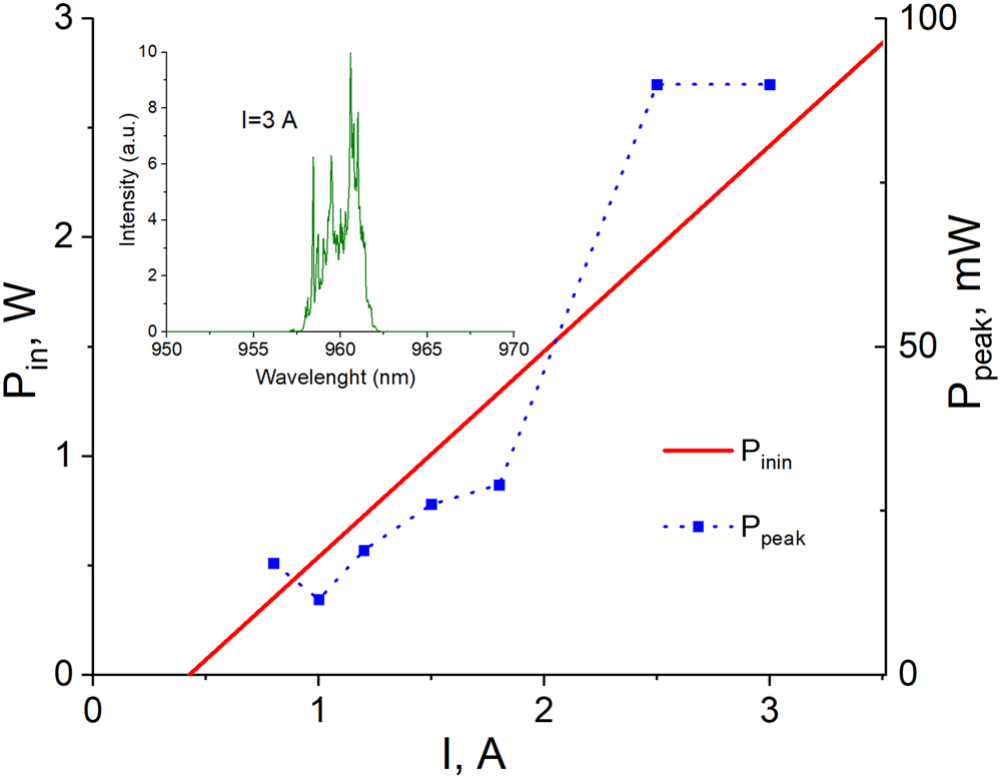
Light-current characteristic of a 960 nm semiconductor laser used in the optical trapping experiments (red line). Blue squares show the optical power in the super-focused needle beam at different pumping levels. Inset shows a typical optical spectrum of a semiconductor laser at pumping current of 3A.

The super-focused laser beam was also produced with the 160° micro-axicon. With this beam we demonstrated optical trapping and tweezing of a rat’s red blood cells. The average size of red blood cells was ~5–6 μm, that better matched with the transverse size of needle beam produced with the 160° micro-axicon rather than with that of 140° counterpart (see Fig. 2). Figure 6 shows the light-current characteristic and typical spectrum of a 960 nm semiconductor laser used in the optical trapping experiments. The laser diode demonstrated the linewidth of few nanometres at all reasonable pumping levels, which is typical for the high-power semiconductor lasers. It is important to note that the poor spectral quality of the laser beam is the limiting factor for its coherence and possibility of self-interference, which is in turn the enabling factor for generation of the Bessel beam. However, we already have demonstrated that generation of Bessel beams is possible with laser diodes of very poor spectral quality and even light-emitting diodes^16^. Also, it was demonstrated that the spatial quality of the laser beam is more important for possibility of generation and quality of Bessel beams rather than the spectral linewidth^21^. Therefore, the demonstration of superfocusing of the multimode laser beam with poor spatial quality is another very important step opening new avenues for direct-diode applications.

**Figure 7:**
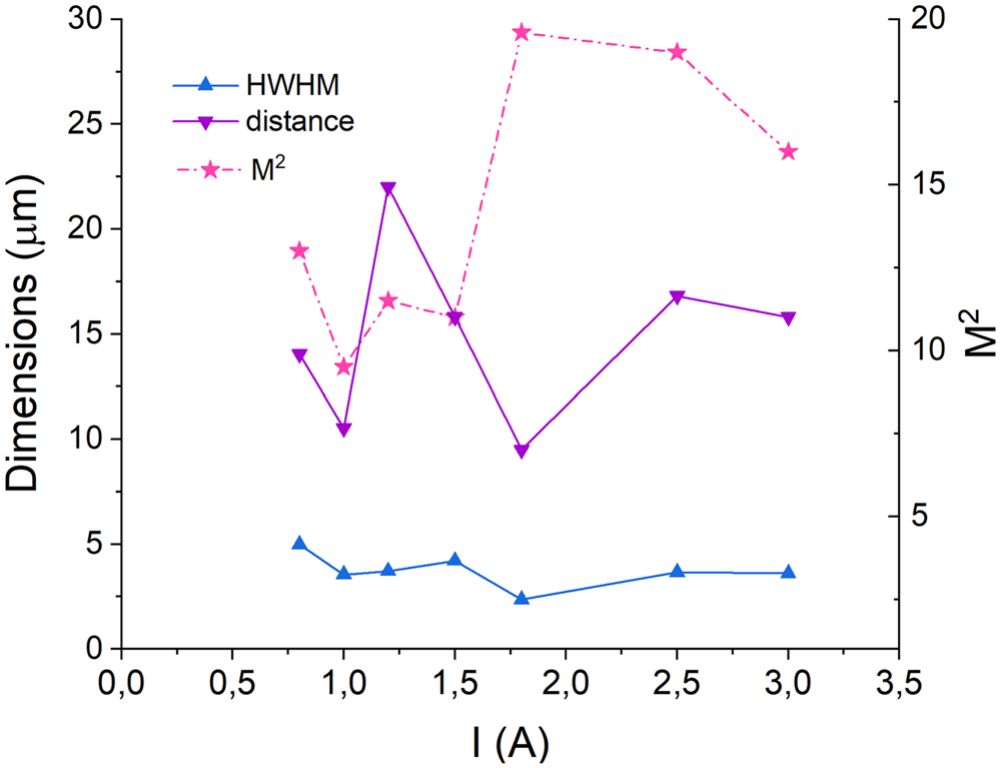
Transverse size (blue up-triangles) and propagation length (violet down-triangles) of the superfocused needle beam generated with 160° microaxicon and a 960 nm semiconductor laser with *M*^2^ factor (pink stars) varying with pump current.

As we mentioned above, the highest power delivered through the fiber axicon in our experiments was near 2.5 W (limited by the laser driver). The focusing process with fiber axicon was highly controllable and stability of the focal region was found to depend solely on the stability of the fiber-coupled laser source. The focalization region originates from the (unrounded) axicon tip and extends through the propagation length defined by the apex angle and aperture of the axicon and parameters of the beam. As seen from Fig. 7, the superfocused beam propagation length was found to be in the range of 10 s μm, while the transverse size was well below 5 μm at all pumping levels. Unfortunately, the laser beam propagation factor M^2^ is difficult to control and variation of the laser power typically results in variation of M^2^. Therefore, no direct comparison of focusing stability at different power levels is possible. However, the performed superfocusing experiments at different power levels demonstrate very reasonable performance and good response stability with respect to the power variation. The peak power in the super-focused needle beam demonstrated nearly linear increase with pumping (shown with blue squares in Fig. 6) tending to 100 mW at pumping of 2.5–3 A. (Here, one should note that the comparison of the optical power in the ‘classical’ focal spot to that in the central lobe of the Bessel beam is not possible as the infinitely long needle beam contains infinitely low power in any cross-section).

**Figure 8:**
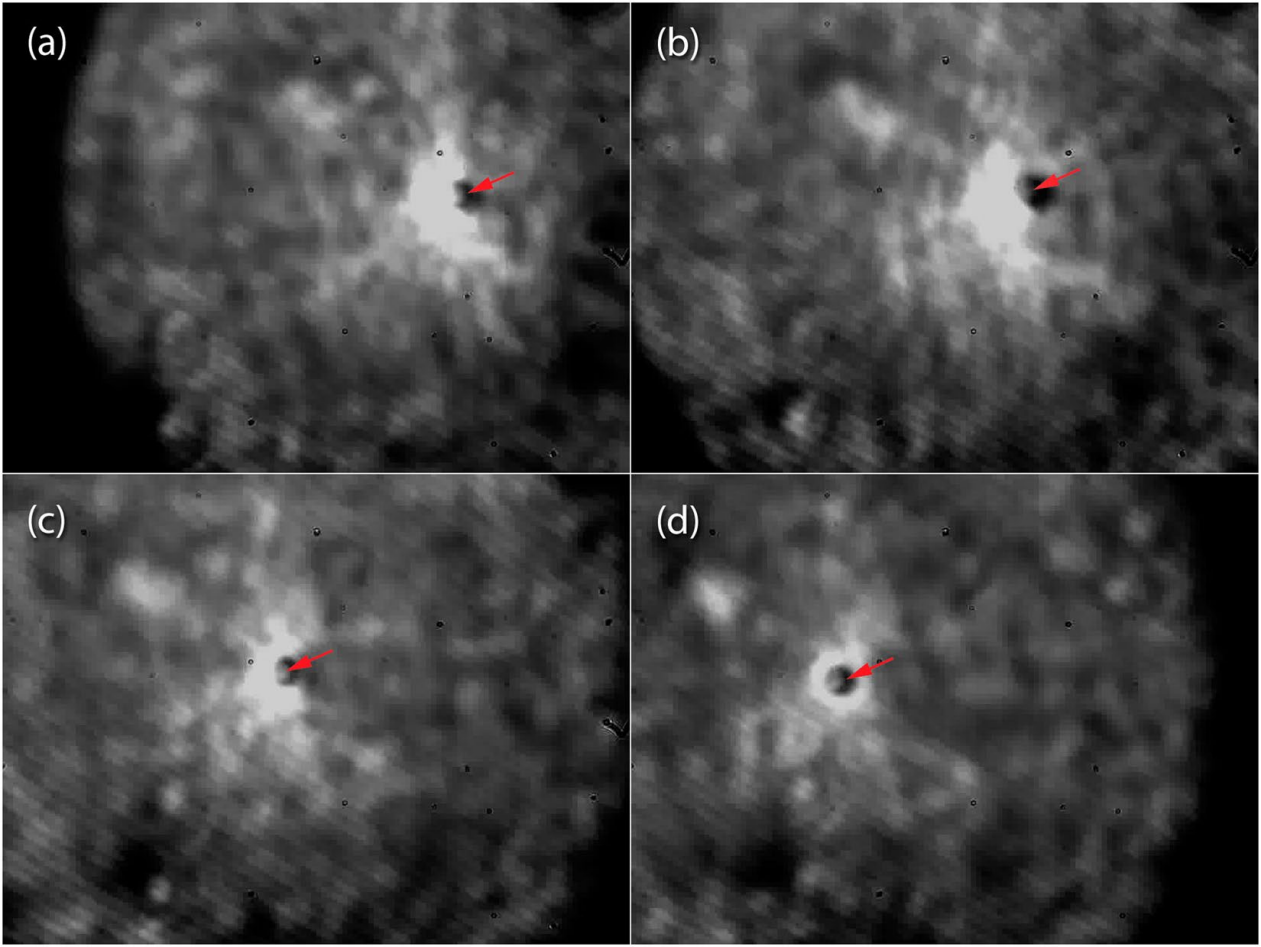
Optical trapping and manipulation of red blood cells with a super-focused beam from a 960 nm semiconductor laser with *M*^2^ = 18. Red arrows indicate the tweezed object and its movement.

In our experiments, we dissolved rat blood in water-heparin solution to ensure the avoidance of clotting. A series of experimental images were reproduced in Fig. 8 that demonstrated the two-dimensional optical trapping and manipulation of red blood cells with the super-focused beam from a high-*M*^2^ semiconductor laser. In this figure, red arrows indicate the tweezed object and its movement. The displacement (the trapping area) was about 100 µm (limited by the field of view of the microscope optical system and CCD camera) and the maximum translation speed was about 10 µm/s. It should be noted that trapping of the red blood cells in our experiments was stable enough to be performed even with non-hydrophobic glass slides. These observations confirm the potential for optical manipulation involving super-focused high-M^2^ laser diodes and raise new opportunities within the applications sector where lab-on-chip configurations can be exploited.

Concerning the optical resistance of the axicons used in our experiments, the highest continuous-wave (cw) power delivered through the fiber axicon was near 2.5 W that translates to the power density of over 0.03 MW/cm^2^. With such power density (limited by the laser driver), no sign of degradation was detected. According to the literature, the results for the photosensitive material (SZ2080 + PI) that had been tested for its laser-induced damage threshold (LIDT)^31^ at the wavelength around 1 μm for series of femtosecond and nanosecond pulses are 0.49 ± 0.05 J/cm^2^ and 10.63 ± 1.07 J/cm^2^ correspondingly. Moreover, another study presenting microlens resiliency to cw and femtosecond pulsed exposure^32^ prove that pure SZ2080 is ∼20 fold more resistant to high irradiance as compared with standard lithographic material (SU8) and can sustain up to 1.91 GW/cm^2^ intensity.

However, probably the most important applications of the demonstrated super-focusing approach seem to be within the direct applications of laser diodes (termed as ‘direct diode’) in materials processing and 3D printing, where both high power and tight focusing is required. Nowadays most such applications rely on diode-pumped solid state lasers (including those with fibre and disc active media). These lasers act as the ‘power-converters’ transforming poor beam-quality of multiple incoherently combined laser diodes into a high-brightness solid state laser beam. Unfortunately, this conversion comes at a cost of significantly reduced wall-plug efficiency and greatly increased foot-print. Therefore, transition to the ‘direct-diode’ and avoiding the detour of having to rely on a solid-state active media has always been thought as the only way for the most efficient conversion of electrical current into the laser light. In this sense, the super-focusing technique, whose immediate application is demonstrated in optical trapping, paves the way for many new ‘direct-diode’ applications and for improvement of the existing ones in soldering, welding, scribing, marking, engraving, paint stripping, powder sintering, synthesis, brazing and machining.

## Conclusion

We believe that our results presented here demonstrate convincingly that super-focused radiation from a semiconductor laser with a high beam propagation parameter can be used in biophotonics applications for optical trapping and tweezing of red blood cells and other microscopic objects. The concept of ‘super-focusing’ for laser-diode high-*M*^2^ beams relies on the generation of a Bessel beam using a conical lens and based on the novel fibre-integrated design for the axicon. We have been able to achieve the transverse size of the needle-beam of approximately 2–4 µm with a propagation length of 20 µm. This was produced with a 140° micro-axicon from a 960 nm laser beam with beam propagation parameter M^2^ = 18. This gives approximately an order of magnitude reduction of the focal spot size in comparison to the minimal focal spot size achievable with such a beam and an ideal NA = 1 lens. These outcomes thus bode well for future diode-based configurations in material processing and 3D laser printing as well as for multiple biomedical photonics-based applications, where both high power and tight focusing is required.

## Methods

The axicon fabrication process exhibited high repeatability. For the 3D laser nano-printing on fibre-end, the axicon was firstly developed using the drawing software SolidWorks® and sliced in 100 nm horizontal slices. Each slice was nano-printed into the photopolymer by moving the focused laser beam using a galvo-scanner (ScanLab). A special base was designed in order to accommodate the fibre and the hybrid material. This base was placed on the x-y-z linear transition PI stage as presented in Fig. 3. In order to align the fiber core with the microscope objective, a He-Ne laser (Uniphase) was coupled to the free end of the fiber. In this way, the light emerging out from the endface of the fiber, which was immersed in the photopolymer, enabled the fiber core to be clearly imaged with the CCD camera and to be aligned to the laser writing beam by moving the linear motion stage (PI). By this way, the axicons of each sample were able to be fabricated concentric to the optical fiber. The beam from a titanium-sapphire femtosecond laser beam (Femtolasers Fusion, 800 nm, 75 MHz, <20 fs) was focused into the organic-inorganic hybrid composite SZ2080^33^ using a microscope objective lens (40×, NA = 0.95, Zeiss, Plan Apochromat). After printing each layer, the sample was lowered 100 nm to “print” the next slice. Movement on the z-axis was carried out using a linear motion stage (PI). The average power used for the fabrication of the axicon was 40 mW measured before the objective (average transmission 20%). The galvo scanning speed was always set at 200 µm/s. After the completion of the laser processing, the unpolymerised SZ2080 was removed by immersion in a 1:1 isopropanol/4-methyl-2-pentanone solution.
